# Insecticidal activity of essential oils from *Piper aduncum* against *Ctenocephalides felis felis*: a promising approach for flea control

**DOI:** 10.1590/S1984-29612024050

**Published:** 2024-09-16

**Authors:** Jeferson Adriano e Silva Assunção, Daniel de Brito Machado, Jessica Sales Felisberto, Douglas Siqueira de Almeida Chaves, Diefrey Ribeiro Campos, Yara Peluso Cid, Nicholas John Sadgrove, Ygor Jessé Ramos, Davyson de Lima Moreira

**Affiliations:** 1 Laboratório de Produtos Naturais e Bioquímica, Diretoria de Pesquisa, Instituto de Pesquisas Jardim Botânico do Rio de Janeiro, Rio de Janeiro, RJ, Brasil; 2 Programa de Pós-graduação em Pesquisa Translacional em Fármacos e Medicamentos, Instituto de Tecnologia em Fármacos, Fundação Oswaldo Cruz – FIOCRUZ, Rio de Janeiro, RJ, Brasil; 3 Departamento de Ciências Farmacêuticas, Instituto de Saúde e Ciências Biológicas, Universidade Federal Rural do Rio de Janeiro – UFRRJ, Seropédica, RJ, Brasil; 4 Departamento de Parasitologia Animal, Instituto de Veterinária, Universidade Federal Rural do Rio de Janeiro – UFRRJ, Seropédica, RJ, Brasil; 5 Roccoco Cosmetics, Yatala, Qld, Australia.; 6 Laboratório Farmácia da Terra, Faculdade de Farmácia, Universidade Federal da Bahia – UFBA, Salvador, BA, Brasil

**Keywords:** Medicinal plants, Piperaceae, flea control, dillapiole, Plantas medicinais, Piperaceae, controle de pulgas, dilapiol

## Abstract

*Piper aduncum* L., a Brazilian medicinal plant, is known for its bioactive properties, including repellent and insecticidal effects. This study investigated the insecticidal potential of essential oils (EOs) from *P. aduncum*, collected during the dry and rainy seasons, against fleas (*Ctenocephalides felis felis* Bouché, 1835) in egg and adult stages. The EOs were obtained by hydrodistillation using a modified Clevenger apparatus for 2 h. Qualitative and quantitative analysis were performed via gas chromatography. The findings revealed that dillapiole was the predominant substance in both EOs, accounting for 77.6% (rainy) and 85.5% (dry) of the EOs. These EOs exhibited high efficacy against the parasite *C. felis felis*, resulting in 100% egg mortality at a concentration of 100 μg/mL and 100% mortality for adult fleas starting from 1,000 μg/mL. Dillapiole standard was also effective but at a relatively high concentration. This finding suggested that EOs from *P. aduncum* exhibit cytotoxicity against these pests and might hold potential for commercial production, offering practical applications for such bioprospecting. This study uniquely revealed that the EOs from *P. aduncum*, which is rich in dillapiole, demonstrated pulicidal activity against the parasite *C. felis felis*, particularly in inhibiting the hatching of the eggs of these parasites.

## Resumo

*Piper aduncum* L., uma planta medicinal brasileira, é conhecida por suas propriedades bioativas, incluindo efeitos repelentes e inseticidas. Este estudo investigou o potencial inseticida dos óleos essenciais (OEs) de *P. aduncum,* coletados na estação seca e chuvosa, contra pulgas (*Ctenocephalides felis felis* Bouché, 1835) em estágios de ovos e adultos. Os OEs foram obtidos por hidrodestilação em aparelho de Clevenger, modificado por 2 horas. Análises qualitativas e quantitativas foram realizadas por meio de cromatografia gasosa. Os resultados revelaram que o dilapiol foi a substância predominante, representando 77,56% (chuvoso) e 85,52% (seco) dos OEs. Esses OEs mostraram alta eficácia contra o parasita *C. felis felis*, resultando em 100% de mortalidade de ovos a uma concentração de 100 μg/mL e 100% de mortalidade para pulgas adultas a partir de 1.000 μg/mL. O padrão dilapiol também foi eficaz, mas em uma concentração relativamente alta. Este achado sugere que os OEs de *P. aduncum* exibem citotoxicidade contra esses parasitas e podem ter potencial para a produção comercial, oferecendo aplicações práticas para essa bioprospecção. Esse estudo revelou de maneira única que os OEs de *P. aduncum*, ricos em dilapiol, demonstraram atividade pulicida contra o parasita *C. felis felis*, inibindo, especialmente, a eclosão dos ovos desses parasitas.

## Introduction

*Piper aduncum* L., a member of the Piperaceae family, is recognized under various names including “Aperta-ruão”, “Matico”, “Pimenta-de-Macaco" and “Pimenta-Longa”. This species is prevalent across diverse Neotropical regions, as highlighted in other studies (Jaramillo & Manos, 2001; Morais et al., 2023). It has been extensively utilized in traditional medicine, holding significant value among local and indigenous communities globally for the treatment of respiratory, gynecological, gastrointestinal ailments, and kidney disorders (Bourdy Bourdy et al., 2000; Siges et al., 2005; Pohlit et al., 2006). Furthermore, its use as an insecticide has been documented in traditional communities within Papua New Guinea (Siges et al., 2005).

The essential oils (EOs) derived from *P. aduncum* exhibit noteworthy bioactive potential. The species yields a variety of essential oil chemotypes, which include notable constituents such as camphor, piperitone, benzaldehyde, asaricine, safrole, apiole, dillapiole, 1,8-cineole, *E*-nerolidol, linalool, β-bisabolene, and β-selinene, all of which support a wide spectrum of biological activities. This extends to antimicrobial properties, as well as acaricidal, antiparasitic, and insecticidal effects against 23 arthropods crucial in agriculture and livestock (Durofil et al., 2021; Morais et al., 2023). The literature describes a high chemical phenotypic plasticity due to biotic and endoclimatic factors, such as rainy and dry periods, which may result in variations in biological activity (Durofil et al., 2021; Morais et al., 2023; Ramos et al., 2023; Assunção et al., 2023). However, research gaps exist in fully assessing the insecticidal efficacy of these EOs against both larval stages and adult individuals of fleas (*Ctenocephalides felis felis*, Bouché, 1835).

Globally, about 2,574 species of flea have been identified, with both males and females engaging in blood-feeding. These ectoparasites are known for their remarkable adaptability, infesting a wide range of hosts including carnivores, rodents, ungulates, birds, and humans (Bitam et al., 2010; Wu et al., 2023). The flea stands out due to its widespread distribution and medical significance, representing a considerable public health concern due to its vector capabilities for transmitting a wide array of pathogenic microorganisms. These include viruses and bacteria such as *Bartonella henselae*, *Rickettsia felis* and *Rickettsia typhi* (Linardi & Santos, 2012; Horta et al., 2014; Wu et al., 2023). The growing resistance of these fleas to synthetic insecticides further complicates control measures (Linardi & Santos, 2012; Horta et al., 2014; Wu et al., 2023). Given the environmental and health concerns associated with synthetic insecticides, EOs present a sustainable alternative for pest management (Liu et al., 2018; Ortega et al., 2018; Zhu et al., 2020; Brito et al., 2021).

This study aims to elucidate the insecticidal activity of essential oils (EOs) from leaves of *P. aduncum* cultivated in southeastern Brazil, collected during the dry and rainy seasons, against *C. felis felis*. Through this research, we seek to promote the utilization of these natural resources, particularly those now in cultivation and available for industry. Furthermore, we aim to promote uses that align with traditional practices.

## Methods

### Plant material and essential oil

Fresh leaves from *Piper aduncum* L. were cultivated for 460 days and harvested for the current study in the reproductive stage during February (rainy season, Sample 1) and November (dry season, Sample 2) during 2022 at 9 a.m., from an agroecological cultivation system located in the Socioenvironmental Responsibility Center of the Rio de Janeiro Botanical Garden, Brazil (coordinates: 22°58'00''S/43°13'43''W, at an altitude of 26 m above sea level). The agroecological cultivation procedure was previously described (Assunção et al., 2023). The species was identified by Prof. Dr. Elsie Franklin Guimarães at the Research Institute of the Rio de Janeiro Botanical Garden (JBRJ), Brazil. A specimen sample (voucher) was deposited in the RB Herbarium of JBRJ, Rio de Janeiro, Brazil (RB01426180). A license for genetic heritage access for research and technological development was obtained from the National System of Genetic Heritage and Traditional Knowledge Management (SisGen) under the number AE20045.

Fresh leaves of *P. aduncum* were subjected to hydrodistillation using a Clevenger apparatus following the standard procedure (Ramos et al., 2022a; Assunção et al., 2023). Initially, 200 g of fresh leaves were weighed for each sample (1 and 2) and manually chopped using scissors. After 2 h of distillation, the EOs were separated from the water layer, dried over anhydrous sodium sulfate (Na_2_SO_4_; Sigma‒Aldrich, Brazil), filtered, and stored at -4°C until analysis.

### GC‒MS and GC‒FID analyses

The EO was diluted in dichloromethane (Merck, Brazil) to a final concentration of 1 mg/mL and then subjected to gas chromatography (GC) coupled to mass spectrometry (GC‒MS) for identification and GC coupled to a flame ionization detector (GC‒FID) for compound quantification. GC‒MS analysis was performed using an HP 6890 GC coupled to an Agilent MS 5973 N mass spectrometer (Hewlett-Packard, Brazil) operating at 70 eV ionization energy in positive mode with a mass range of 40–600 m/z. GC was performed with an HP-5MS capillary column (30 m × 0.25 mm I.D. × 0.25 μm film thickness), with helium (~99.999%) serving as the carrier gas at a constant flow rate of 1.0 mL/min. The temperature program ranged from 60°C to 240°C, increasing at 3°C/min. The injector and detector temperatures were set at 270°C, and 1 µL samples were injected in split-less mode (Ramos et al., 2022a; Assunção et al., 2023).

Quantitative data on volatile constituents were obtained by normalizing peak areas using a GC‒FID HP-Agilent 6890 (Hewlett-Packard, Brazil) operating under the same conditions as those used for GC‒MS. The retention index (RI) and peak area quantification were obtained based on the GC‒FID results. The relative percentage of individual components was calculated based on GC peak areas without FID response factor correction. RIs were calculated for separated compounds relative to n-alkanes (C_8_-C_28_; Sigma‒Aldrich, Brazil) (van Den Dool & Kratz, 1963). The constituents were identified by comparing their calculated RIs with literature values and by comparing their mass spectra with those registered by the National Institute of Standards and Technology (NIST14) and ChemStation Data System (WILEY7n) libraries (Adams, 2007). Additionally, co-injection of authentic standards was performed, especially for the major compound dillapiole (BCCH5270, 99.7% purity; Sigma‒Aldrich, Brazil).

### Insecticidal activity

The insecticidal activity of the EO from fresh leaves of *P. aduncum* was tested against eggs and adult cat fleas at the Laboratory of Bioactive Natural Chemistry at Federal Rural University of Rio de Janeiro (UFRRJ).

### Preparation of dilutions

For the preparation of various concentrations of the EOs, pure acetone (HPLC grade, Merck, Brazil) was chosen as the solvent. To evaluate the insecticidal activity, filter paper strips (Whatman No. 1, 80 g) with an area of 10 cm^2^ (1 × 10 cm) were saturated with 0.200 mL of each EO solution. The tested concentrations ranged from 250, 500, 1000, 2000, 4000, 8000, 10000, 12000, and 15000 µg/mL. After the impregnation of the filter paper strips, this concentration range corresponded to 5 to 160 µg/cm^2^ of EO on the filter paper strips.

To evaluate the hatching inhibition activity of *C. felis felis* eggs, filter paper discs with an area of 23.7 cm^2^ were used, which were saturated with 0.470 mL of each EO solution. The tested concentrations ranged from 10 to 1,000 µg/mL, equating to 0.2 to 20 µg/cm^2^ post-impregnation of EO.

Moreover, filter paper strips and discs saturated with the analytical standard dillapiole (BCCH5270, 99.7% purity, Sigma-Aldrich, Brazil) were used at the same concentration as the tests conducted with EOs from *P. aduncum* (Oliveira et al., 2022; Soares et al., 2023).

### Determination of biological activity

To determine the EO activity against adult fleas, ten 14-day-old unfed individuals, comprising five males and five females for each replicate, were selected. The insects were placed in a test tube (1 × 10 cm) with filter paper strips impregnated with different concentrations of EOs, as described in the previous section. After the fleas were placed in test tubes, the materials were incubated in climate-controlled chambers at a controlled temperature and relative humidity (27 ± 1°C; 75 ± 10%). The tests lasted for 24 h and 48 h. The motility criterion used was movement, where any minimal movement exhibited by the insect was considered indicative of life (Oliveira et al., 2022; Soares et al., 2023).

For the analysis of the inhibition of the biological cycle of *C. felis felis*, 10 eggs younger than 24 h were placed in plastic Petri dishes (60 × 15 cm) containing filter paper discs impregnated with different concentrations of EO, as described in the previous subsection. Shortly after, half a gram of specific larval development diet, as described by Correia et al. (2003), was added. The Petri dishes were closed with lids and placed in a climate chamber at a temperature of 27 ± 1°C and a relative humidity of 75 ± 10% for a period of 30 days. Any egg that failed to generate an adult flea was considered non-viable (Oliveira et al., 2022; Soares et al., 2023).

As negative controls for the tests, both the filter paper discs and strips were not impregnated with EO. The negative control assessed the viability of the manipulated fleas during the tests. For the placebo, filter paper strips and discs impregnated with the same volume of pure acetone, but no EO, were tested in tandem with the treatment groups. Fipronil was used as a positive control to assess activity against adult fleas at a concentration of 400 µg/mL (equivalent to 8 µg/cm^2^). Piriproxifen was used as a positive control for the inhibition of the biological cycle at the same concentration as fipronil. All tests were carried out in sextuplicate (six replicates of ten individuals or eggs each). After the assessments, the data were tabulated, and the mortality percentage was calculated for each concentration (Oliveira et al., 2022; Soares et al., 2023).

### Data analysis

In the insecticidal activity tests, after counting the number of live and dead individuals, the mortality percentage was calculated for each concentration using the formula described by Abbott (1925). The experimental data were tabulated, and the lethal concentration values (LC_50_) for each treatment were statistically calculated using probit analysis conducted with R Studio Team software (2020, R Studio: Integrated Development Environment for R. RStudio, PBC, Boston, MA, USA), with a statistical significance set at 5% (*p* < 0.05).

## Results and Discussion

### Chemistry of essential oils

Hydrodistillation of leaves from *Piper aduncum* yielded milky-colored EOs characterized by a pleasantly spicy and pungent aroma. GC-MS analysis identified a majorly dominant volatile compound in the EO exorcised during both months, February and November 2022 (the rainy season, sample 1, and the dry season, sample 2, respectively). Notably, sample 1's yield was 1.06% ± 0.3%, with an arylpropanoid being the predominant constituent (77.7%), followed by non-oxygenated monoterpenes (8.3%) and sesquiterpenes (8.0%). A total of 41 components were identified, accounting for 96.3% of the total components.

**Figure 1:**
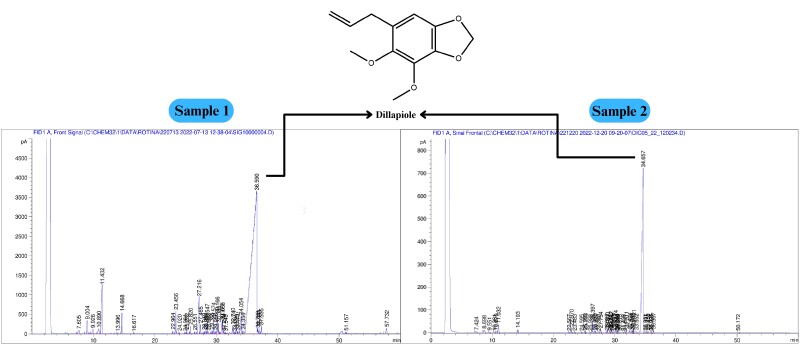
Volatile substances from *Piper aduncum* identified through gas chromatography coupled to mass spectrometry (GC‒MS) analysis and quantified using gas chromatography coupled to a flame ionization detector (GC‒FID).

In contrast, sample 2 exhibited a higher yield of 3.0% ± 0.1%, with a higher arylpropanoid content of 85.5% (Figure 1). Non-oxygenated monoterpenes (4.0%) and non-oxygenated sesquiterpenes (3.3%). A smaller amount of oxygenated sesquiterpenes (0.7%) was also identified. A total of 10 components were identified, representing 93.5% of the total components.

Dillapiole was the major constituent identified in both the samples, comprising 77.6% in sample 1, and 85.5% in sample 2 (Table 1). This is consistent with several earlier studies conducted on *P. aduncum* specimens collected from different regions of Brazil, all of which identified dillapiole as the primary constituent, with a high yield of essential oil (EO). For instance, specimens obtained from Northern Brazil, Amapá State (97.3%), Amazonas State (85.6%), Pará State (88.9%), and Northeast Brazil, Pernambuco State (96.9%), all yielded highly pure dillapiole. Conversely, dillapiole was detected in a sample from Brasília (Central Brazil region) but at a lower relative percentage (49.5%) (Maia et al., 1998; Sousa et al., 2008; Potzernheim et al., 2012; Silva et al., 2013; Ramos et al., 2022a). All earlier studies focused on wild specimens of *P. aduncum*, while the current study is the first to confirm a consistent chemical profile from a cultivated specimen in the State of Rio de Janeiro, Brazil. The significance of this is illustrated in another study of cultivated species of *Piper*, where chemical profiles change dramatically when propagated in different soil types (Ramos et al., 2022b).

**Table 1:** Chemical composition and yields of essential oils from the leaves of *Piper aduncum*.

Compound*	**RI_lit_**	**RI _calc_**	**% ± SD**	
			**Sample 1**	**Sample 2**
α-pinene	932	935	0.52±0.01	0.37±0.01
β-pinene	974	977	1.71±0.01	1.07±0.01
myrcene	988	989	tr	-
α-phellandrene	1002	1005	0.47±0.01	-
limonene	1024	1027	tr	-
β-phellandrene	1025	1029	tr	-
*Z*-β-ocimene	1032	1035	0.57±0.01	-
*E*-β-ocimene	1044	1047	3.72±0.01	0.21±0.01
γ-terpinene	1054	1050	-	2.33±0.01
terpinolene	1086	1088	tr	-
linalool	1095	1099	tr	-
*allo*-ocimene	1128	1128	1.09±0.01	-
δ-elemene	1335	1335	tr	-
cyclosativene	1369	1370	0.06±0.01	-
α-ylangene	1373	1376	0.10±0.01	-
β-elemene	1389	1390	0.40±0.01	-
α-gurjunene	1409	1408	0.09±0.01	-
*E*-caryophyllene	1417	1420	3.07±0.01	1.71± 0.01
γ-elemene	1434	1430	0.21±0.01	-
α-humulene	1452	1456	0.57±0.01	-
*allo*-aromadendrene	1458	1461	0.13±0.01	-
*E*-cadinene-1(6),4-diene	1475	1473	tr	-
germacrene D	1480	1479	1.07±0.01	0.36±0.01
γ-amorphene	1495	1494	0.46±0.01	-
bicyclogermacrene	1500	1497	0.87±0.01	0.52±0.01
δ-amorphene	1511	1509	0.71±0.01	-
γ-cadinene	1513	1514	tr	-
δ-cadinene	1522	1517	tr	0.66±0.01
*trans*-cadinane-1,4-diene	1533	1534	tr	-
elemicin	1555	1551	tr	-
*E*-nerolidol	1561	1562	tr	-
palustrol	1567	1570	tr	-
germacrene D-4-ol	1574	1578	0.60±0.01	-
caryophyllene oxide	1582	1584	tr	-
globulol	1590	1591	tr	0.73±0.01
viridiflorol	1592	1596	1.31±0.01	-
ledol	1602	1605	0.12±0.00	-
dillapiole	**1620**	**1622**	**77**.**56±0**.**01**	**85.52±0.06**
4,6-dimethoxy-5-vinyl-1,2-benzodioxole	1653	1654	tr	-
*epi*-β-bisabolol	1670	1672	tr	-
apiol	1677	1680	tr	-
*n*-eicosane	2000	1998	0.51±0.00	-
**Non-oxygenated monoterpenes**			8.28	3.98
**Oxygenated monoterpenes**			0.05	-
**Non-oxygenated sesquiterpenes**			7.99	3.25
**Oxygenated sesquiterpenes**			2.32	0.73
**Arylpropanoids**			**77.66**	**85.52**
**Number of Identified Compounds**			**41**	**10**
**Total Compounds Quantified (%)**			**96**.**30±0**.**03**	**93**.**49±0**.**07**
**Essential Oil Yield (%)**			**1**.**06±0**.**3**	**3.04±0**.**1**

**Legend.** RI_calc_ = calculated retention index (column HP-5MS, see experimental); RI_lit_ = literature retention index (Adams, 2007); SD = standard deviation from three experimental replicates; % = relative percentage area.

* = all substances were identified by their mass spectra and RI comparison (see experimental).

OEs = essential oils; major compounds are highlighted in bold; tr = trace (percentage value equal to or less than 0.05%), - = substance not detected. Sample 1 - EO extracted in February 2022; Sample 2 - EO extracted in November 2022.

Potzernheim et al. (2012) demonstrated variations in the percentage of EOs (w/w) from four distinct populations of *P. aduncum*, ranging from 0.6% to 1.3%. According to the authors, these yields fall within expectations for commercial production. Yield variations in EOs are commonplace in the species from the genus *Piper* L. For instance, Schindler et al. (2018) observed higher EO yields in the leaves (1.6%) of *Piper gaudichaudianum* Kunth during the spring and lower yields (1.3%) during the summer.

In addition to seasonal variations, there are also variations in EO yields related to the time of day, as noted by Ramos et al. (2020). The authors analyzed the circadian rhythm of EOs from the leaves of *Piper mollicomum* Kunth and identified yield variations ranging from 0.2% to 2.9%, with collections taking place in March and from 1.0% to 3.3% in October. Brito-Machado et al. (2022) evaluated the variation of terpenes in EOs from different reproductive stages of *P. mollicomum*. The percentage of certain volatile constituents, such as limonene, 1,8-cineole, linalool, and eupatoriochromene, increased during the maturation period of the inflorescences and decreased during the fruiting period, suggesting defensive activities against herbivores and attraction of pollinators.

The EOs yields of *P. aduncum* determined in this study, which were 1.1% and 3.0%, are consistent with yields from other commercially marketed species. For instance, the EO yield of *Matricaria chamomilla* L. (Asteraceae) varies between 0.4% and 0.9%, while the EO yield of *Varronia curassavica* Jacq. (Cordiaceae) ranges from 0.4% to 2.7% (Marques et al., 2019; Mavandi et al., 2021).

It is important to emphasize that these variations are common in medicinal plants in general and have a direct impact on their biological effects in humans and from an ecological perspective (Ramos et al., 2023). Therefore, this *P. aduncum* accession achieves a chemical standard that makes it suitable for commercial production.

### Inhibition of *C. felis felis*

The EOs obtained from samples 1 and 2, containing 77.6% and 85.5% of dilapiol respectively, showed good *in vitro* efficacy, resulting in 100% mortality of eggs when applied at a concentration of 100 μg/mL. The analytical standard of dillapiole at 99.7% purity was also effective but required a higher concentration of 1,000 μg/mL to achieve the same level of inhibition. Alternatively, adult parasites were completely inhibited when exposed to higher concentrations of EOs and dillapiole. The differences in lethal doses between the two life stages may be attributed to the increased resistance of adults to insecticides (Rust, 2020).

For sample 1, the perceived mortality rate reached 100% at a concentration of 4,000 μg/mL (after 24 hours of incubation) but after 48 hours of incubation the result changed to 8,000 μg/mL, as shown in Table 2. Because the criterion for mortality was based on perceived movement of the fleas, this illustrates that some individuals were inhibited or immobilized, rather than deceased, after 24 hours of exposure to the EO.

**Table 2:** Effect of treatment with different concentrations of essential oil from *Piper aduncum* L., extracted in February 2022, on flea mortality rates and LC_50_ at different life stages.

**Essential oil with 77.56% Dillapiole**											
**Eggs (30 days)**				**Adults (24 hours)**				**Adults (48 hours)**			
**µg/mL**	**µg/cm^2^**	**Total incubated eggs**	**Mortality (%)**	**µg/mL**	**µg/cm^2^**	**Total incubated fleas**	**Mortality (%)**	**µg/mL**	**µg/cm^2^**	**Total incubated fleas**	**Mortality (%)**
Placebo	- - -	60	0.00	Placebo	- - -	60	0.00	Placebo	- - -	60	0.00
Control (-)	- - -	60	0.00	Control (-)	- - -	60	0.00	Control (-)	- - -	60	0.00
10	0.2	60	11.11	250	5.00	60	20.00	250	5.00	60	15.00
25	0.5	60	24.07	500	10.00	60	56.67	500	10.00	60	38.33
50	1.0	60	53.70	1000	20.00	60	61.67	1000	20.00	60	48.33
75	1.5	60	77.78	2000	40.00	60	90.00	2000	40.00	60	71.67
100	2.0	60	100.00	4000	80.00	60	100.00	4000	80.00	60	83.33
150	3.0	60	100.00	6000	120.00	60	100.00	6000	120.00	60	93.33
250	60	60	100.00	8000	160.00	60	100.00	8000	160.00	60	100.00
500	80	60	100.00	10000	200.00	60	100.00	10000	200.00	60	100.00
1.000	100	60	100.00	12000	240.00	60	100.00	12000	240.00	60	100.00
-------	-------	-------	-------	15000	300.00	60	100.00	15000	300.00	60	100.00
LC_50_ = 0.6 μg/cm^2^				LC_50_ = 10.94 μg/cm^2^							
Minimum – Maximum = 0.5 – 0.8 μg/cm^2^				Minimum – Maximum = 8.14 – 13.95 μg/cm^2^							
Slope = 2.40				Slope = 2.31							
R^2^ = 0.999				R^2^ = 0.859							
*p* = 1.000				*p* = 0.977							

For sample 2, a concentration of only 1,000 μg/mL was necessary for both incubation periods (24 and 48 hours), as detailed in Table 3. Regarding the dillapiole analytical standard, a 100% mortality rate was achieved at a concentration of 7,000 μg/mL for both incubation times (24 and 48 hours), with detailed results available in Table 4. As expected, the blank sample and the negative control did not result in mortality at any stage of the parasite life cycle. These findings are significant for both life cycles of *C. felis felis*, demonstrating that a low concentration of EO can lead to a high mortality rate, particularly by inhibiting egg hatching.

**Table 3:** Effect of treatment with different concentrations of essential oil from *Piper aduncum* L., extracted in November 2022, on flea mortality rates and LC_50_ at different life stages.

**Essential oil with 85.52% Dillapiole**											
**Eggs (30 days)**				**Adults (24 hours)**				**Adults (48 hours)**			
**µg/mL**	**µg/cm^2^**	**Total incubated eggs**	**Mortality (%)**	**µg/mL**	**µg/cm^2^**	**Total incubated fleas**	**Mortality (%)**	**µg/mL**	**µg/cm^2^**	**Total incubated fleas**	**Mortality (%)**
Placebo	- - -	60	0.00	Placebo	- - -	60	0.00	Placebo	- - -	60	0.00
Control (-)	- - -	60	0.00	Control (-)	- - -	60	0.00	Control (-)	- - -	60	0.00
10	0.2	60	14.29	100	2.00	60	6.67	100	2.00	60	6.67
25	0.5	60	35.71	250	5.00	60	33.33	250	5.00	60	33.33
50	1.0	60	67.86	500	10.00	60	61.67	500	10.00	60	61.67
75	1.5	60	85.71	750	15.00	60	86.67	750	15.00	60	86.67
100	2.0	60	100.00	1000	20.00	60	100.00	1000	20.00	60	100.00
150	3.0	60	100.00	1250	25.00	60	100.00	1250	25.00	60	100.00
250	60.0	60	100.00	2500	50.00	60	100.00	2500	50.00	60	100.00
500	80.0	60	100.00	5000	100.00	60	100.00	5000	100.00	60	100.00
1.000	100	60	100.00	10000	200.00	60	100.00	10000	200.00	60	100.00
-------	-------	-------	-------	15000	300.00	60	100.00	15000	300.00	60	100.00
LC_50_ = 0.5 μg/cm^2^				LC_50_ = 6.72 μg/cm^2^							
Minimum – Maximum = 0.4 – 0.7 μg/cm^2^				Minimum – Maximum = 5.46 – 8.03 μg/cm^2^							
Slope = 2.60				Slope = 3.28							
R^2^ = 0.984				R^2^ = 0.988							
*p* = 0.956				*p* = 0.983							

**Table 4:** Effect of treatment with different concentrations of the analytical standard dillapiole on flea mortality rates and LC_50_ at different life stages.

**Dillapiole at 99.70%**											
**Eggs (30 days)**				**Adults (24 hours)**				**Adults (48 hours)**			
**µg/mL**	**µg/cm^2^**	**Total incubated eggs**	**Mortality (%)**	**µg/mL**	**µg/cm^2^**	**Total incubated fleas**	**Mortality (%)**	**µg/mL**	**µg/cm^2^**	**Total incubated fleas**	**Mortality (%)**
Placebo	0	60	0.00	Placebo	0	60	0.00	Placebo	- - -	60	0.00
Control (-)	0	60	0.00	Control (-)	0	60	0.00	Control (-)	- - -	60	0.00
100	2	60	14.29	1000	20	60	13.33	1000	20	60	13.33
250	5	60	26.79	2000	40	60	36.67	2000	40	60	36.67
500	10	60	41.07	3000	60	60	48.33	3000	60	60	48.33
750	15	60	71.43	4000	80	60	68.33	4000	80	60	71.64
1000	20	60	100.00	5000	100	60	80.00	5000	100	60	78.18
2000	40	60	100.00	6000	120	60	90.00	6000	120	60	90.00
3000	60	60	100.00	7000	140	60	100.00	7000	140	60	100.00
4000	80	60	100.00	8000	160	60	100.00	8000	160	60	100.00
5000	100	60	100.00	9000	180	60	100.00	9000	180	60	100.00
-------	-------	-------	-------	10000	200	60	100.00	10000	200	60	100.00
LC_50_ = 7.0 μg/cm^2^				LC_50_ = 51.71 μg/cm^2^							
Minimum – Maximum = 5.3 – 8.9 μg/cm^2^				Minimum – Maximum = 44.5 –58.7 μg/cm^2^							
Slope = 2.20				Slope = 3.30							
R^2^ = 0.993				R^2^ = 0.992							
*p* = 1.000				*p* = 0.888							

The LC_50_ values for eggs were lower than those for adults, indicating that eggs are more susceptible to EO treatment. Notably, the EO containing 85% dillapiole (sample 2) had a lower LC_50_ for eggs (0.5 μg/cm^2^) and adults (6.72 μg/cm^2^) compared to the EO with 77% dillapiole (sample 1 - eggs, 0.6 μg/cm^2^; adults, 10.94 μg/cm^2^). While this suggests that a higher dillapiole content may enhance the EOs efficacy against the parasite, in line with Rust (2020), the activity of pure dillapiole contradicts this, indicating that the interpretation is more complex.

The dillapiole analytical standard exhibited insecticidal activity against both eggs and adult *C. felis felis*. However, its LC_50_ was higher than that of the *P. aduncum* EOs, at 7.0 μg/cm^2^ for eggs and 51.71 μg/cm^2^ for adults. This implies that the EO might be more effective than the dillapiole analytical standard alone in eliminating the parasite. These findings indicate that the EOs of *P. aduncum* show significant insecticidal activity against *C. felis felis*, outperforming the isolated compound.

It is indeed worth noting that sample 2, whose EO has a higher concentration of dillapiole, exhibited a greater pulicidal action than sample 1 and the analytical standard, as well as an ovicidal action equal to sample 1, demonstrating superiority in terms of insecticidal effect for the prospect of a commercial product for external use. However, this needs to be interpreted further, because it is feasible that additive or synergistic effects occur in combination with the other components of the EO.

Hence, while the insecticidal potential of *P. aduncum* EO can be attributed to dillapiole, the other chemical constituents such as myristicin, 1,8-cineole, and β-ocimene have demonstrated efficacy against the same organism in previous studies (Oliveira et al., 2013; Bernuci et al., 2016; Volpe et al., 2016; Cossolin et al., 2019).

Dillapiole is recognized as a primary bioactive compound in *P. aduncum* EO, showing effects against 23 arthropods of agricultural and veterinary importance (Durofil et al., 2021). The study by Durofil et al. (2021) also highlighted the EO's activity against vectors of zoonotic diseases, such as *Aedes aegypti* and *Aedes albopictus*, as well as against etiological agents, such as *Leishmania amazonensis* and *Plasmodium falciparum* (Misni et al., 2011; Parise-Filho et al., 2011; Silva et al., 2019). Nevertheless, the insecticidal property of EO from *P. aduncum* means that at lower a concentration it acts as a repellent, offering promising results against not only fleas, but also the agricultural pests like the spider mite *Tetranychus urticae* and the red flour beetle *Tribolium castaneum* (Jaramillo-Colorado et al., 2015; Araújo et al., 2020).

It should be noted that there are some limitations to the use of EOs in pets, such as dogs and cats. These limitations hinder their broader application, mainly due to some inherent negative properties, such as instability. In certain cases, EOs can be quickly deactivated after application (Turek & Stintzing, 2013). Additionally, although EOs are generally considered safe for human medicinal use, they present toxicity risks for pets, potentially linked to the peculiarities of their metabolism (Štrbac et al., 2021). For this reason, it is recommended that applications be made in more suitable formulations, such as common liquid sprays or nanostructured formulations, for example, nanoemulsions and microemulsions.

In conclusion, to gain acceptance in industry it is necessary to conduct further studies on the safety and efficacy of formulations based on the EO of *P. aduncum*, specifically chemotypes that are rich in dillapiole. The pantropical distribution, ease of cultivation, and high production capacity of this EO makes it a worthy candidate for further development for industry, particularly where pets and people can make use of it as a nature-based alternative to the synthetic products already available in the marketplace. The current study highlights for the first time the good pulicidal activity of EO from *P. aduncum* against *C. felis felis*, demonstrating effectiveness even at low concentrations, making it a viable return on investment. Serums formulated to contain a mere 0.1 – 0.2% can reduce flea infestation by interrupting the reproductive cycle, or at a higher concentration of 1% it can cause direct contact inhibition and mortality. These findings also encourage further research in the field of natural alternatives to synthetics, with the objective of finding more biologically acceptable forms of pest control.
